# Supplementary material for: Ultra-Sensitive and Semi-Quantitative Vertical Flow Assay for the Rapid Detection of Interleukin-6 in Inflammatory Diseases
Source: Biosensors (Basel). 2022 Sep 14;12(9):756. doi: 10.3390/bios12090756 (PMC9496834; doi:10.3390/bios12090756)
Supplement: Supplementary file 1 [file biosensors-12-00756-s001.zip › biosensors-1845402-supplementary.pdf]

Supporting Information

Ultra-Sensitive and Semi-Quantitative Vertical Flow Assay for the Rapid Detection of Interleukin-6 in Inflammatory Diseases

Rongwei Lei, Hufsa Arain, Maryam Obaid, Nivriti Sabhnani, Chandra Mohan *

Department of Biomedical Engineering, University of Houston, Houston, TX 77004, USA
* Correspondence: cmohan@central.uh.edu; Tel.: +1-713-743-3709

Supplementary Methods:

Assay protocol-1. In total, 80 μL of buffer-1 (20 mM Tris, 150 mM NaCl, 1% BSA, 0.18% Tween-20, 0.5% PEG, pH 8, filtered) was applied to the nitrocellulose membrane (NCM), then 80 μL of blocking buffer (3mM EDTA, 3% BSA, 0.1% gelatin, filtered) was added to NCM for 5 min. Following an additional 80 μL wash with buffer-1, 100 μL of buffer-1 spiked with the analyte or non-spiked (0 ng/mL) was loaded onto two spotted quadrants, respectively. Following an 80 μL wash with buffer-1, 20 μL of 10 μg/mL biotinylated anti-IL-6 in buffer-1 was individually loaded to the two quadrants in NCM. Following an additional 80 μL wash with buffer-1, 5 μL OD = 2 150-SA-GNP in buffer-1 was loaded to the two quadrants, respectively. After 30 s, 800 μL of buffer-1 was loaded to wash off any background.

Assay protocol-2. Buffer-2 (20 mM Tris, 150 mM NaCl, 1% BSA, 0.18% tween 20, 0.5% PVP40, pH 8, filtered) was substituted for buffer-1 in Assay protocol-1 and the following steps remained constant.

Table S1. The mean levels of IL-6 reported in blood, serum, or plasma of healthy controls and various inflammatory diseases.

Condition	Mean IL-6 levels (range)	References
Healthy control	5.2 pg/mL (0-7)	[13]
COVID-19 induced CRS†	40 pg/mL (34.5-214.5)	[14]
Irritable bowel syndrome	1.1 pg/mL (0.5-1.6)	[15]
Obstructive Sleep Apnea	1.2 pg/mL (1.1-1.4)	[16]
Fibromyalgia Syndrome	134.9 pg/mL (67.6-202.2)	[17]
Rheumatoid Arthritis	35.1 pg/mL (13.8-56.4)	[18]
Atrial Fibrillation	14 pg/mL (0.2–156.5)	[19]
Atypical Depression	2.5 pg/mL (1.2-3.8)	[20]
Breast Cancer	111.4 pg/mL	[21]
Sepsis	3.5 ng/mL (<0.1-305)	[22]
COVID-19 Pneumonia	1000 pg/mL (20-10,000)	[23]

CAR-T cell therapy induced CRS†	3000 pg/mL	[24]
Lung cancer (Stage 4)	36.5 pg/mL (20-400)	[25]
Pneumonia	2852 pg/mL	[26]
Polycystic ovary syndrome	4.8 pg/mL (0-1000)	[27]
Ovarian cancer	55.6 pg/mL (0-2869)	[28]
Renal cell carcinoma	224 pg/mL (62-707)	[29]
Pancreatic cancer	3-156 pg/mL	[5]

CRS: Cytokine release syndrome

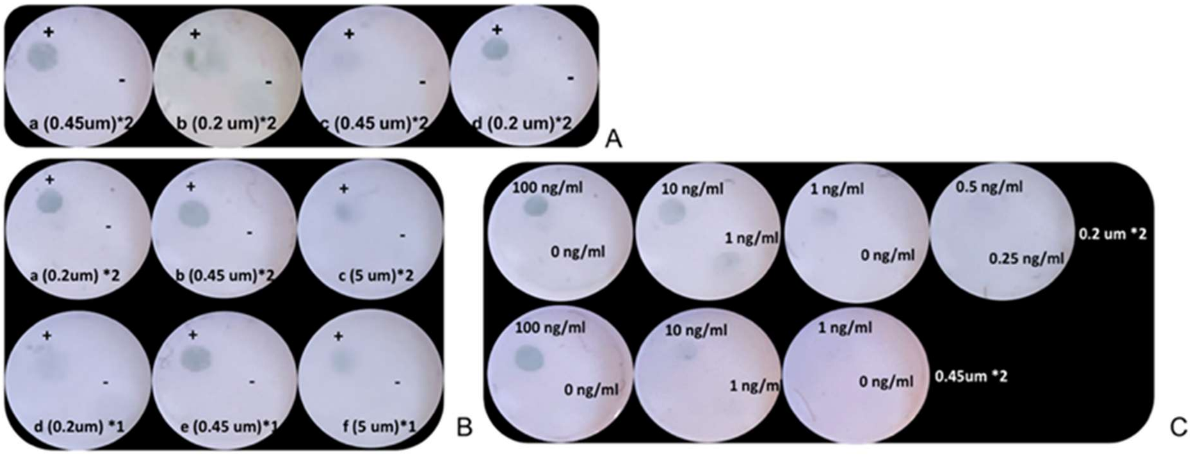

Figure S1. The evaluation of buffer, membrane type, and membrane layer numbers on the performance of 150 nm streptavidin GNP-based (150-SA-GNP) IL-6 VFA. (**A**) Two layers of Cytiva NCM 0.2 µm or 0.45 µm were assembled into the VFA. Each label within the figure displays the pore size and the number of layers of membrane tested. The top left quadrant is marked as + (spiked with 100 ng/mL rIL-6) and the bottom right quadrant is marked as—(non-spiked sample). The + (100 ng/mL) zone should appear as a homogenous, uniform green spot with maximum intensity, while the—(0 pg/mL) test zone should show no signal. The 0.45 µm pore size NCM outperformed 0.2 µm NCM using supplementary methods Assay protocol-1 (**a**,**b**) and 0.2 µm pore size NCM outperformed 0.45 um NCM using supplementary methods Assay protocol-2 (**c**,**d**). (**B**) The first row depicts three types of porous membranes (0.2 µm, 0.45 µm, and 5 µm) that were evaluated to detect 100 ng/mL rIL-6 (+) and non-spiked sample (−) using double-stacked layers of membrane (**a**–**c**). The second row depicts the same three types of porous membranes that were evaluated to detect 100 ng/mL rIL-6 (+) and non-spiked sample (−) using single-stacked layers of membrane (**d**–**f**). The 0.2 µm NCM showed stronger positive signal when using two layers of NCM, while 0.45 µm and 5 µm NCM showed a comparable positive signal with either one or two layers of NCM. (**C**) The first row depicts images corresponding to IL-6 serial dilution, assayed using 0.2 µm NCM, following assay protocol-2. The second row depicts images corresponding to IL-6 serial dilution, assayed using on 0.45 µm NCM using assay protocol-1. Amersham 0.2 µm membrane showed higher sensitivity than Amersham 0.45 µm.

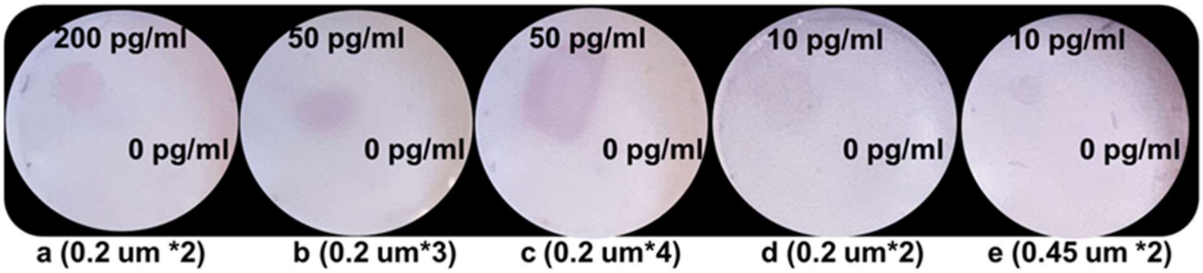

Figure S2. Characteristics of 40 nm streptavidin GNP-based (40-SA-GNP) VFA. Given the limited sensitivity of 150-SA-GNP, 40-SA-GNP was next evaluated. In total, 1 μL of 1 mg/mL anti-IL-6 was spotted on top left quadrant (+pg/mL of rIL-6) and lower right quadrant (0 pg/mL) on 0.2 μm NCM (Cytiva). Two (**a**), three (**b**), and four (**c**) layers of 0.2 μm NCM (Cytiva) were assembled with two layers of CF5 and one layer of CF7 absorbent pad. Following supplementary methods assay protocol-2, rIL-6-spiked buffer-2 (200 pg/mL or 50 pg/mL) and non-spiked (0 ng/mL) were loaded to the top left quadrant and lower right quadrant, respectively. It is found that the position of test zone shifted dramatically when using three NCM layers, while a wide area of positive signal appeared when using four NCM layers, thus rendering two NCM layers a more optimal choice. Following supplementary methods assay protocol-2, two layers of 0.2 μm NCM (**d**), and two layers of 0.45 μm NCM (**e**) were assembled, and both showed a dim signal at the top left quadrant of NCM for detecting 10 pg/mL of rIL-6 and no background at the lower right quadrant of NCM for detecting non-spiked buffer-2 (0 pg/mL).
